# Plasma phosphorylated tau 217 and neurofilament light chain on the association between depressive symptoms and cognitive decline: The Shanghai Aging Study

**DOI:** 10.1017/S0033291725103115

**Published:** 2026-01-20

**Authors:** Wanyu Xia, Chengyin Xu, Zhenxu Xiao, Xiaowen Zhou, Qianhua Zhao, Ding Ding, Wei Deng

**Affiliations:** 1 Department of Biostatistics, School of Public Health, Fudan University, Shanghai 200032, China; 2 Key Laboratory of Public Health Safety, Ministry of Education, Fudan University, Shanghai 200032, China; 3 NHC Key Laboratory of Health Technology Assessment, Fudan University, Shanghai 200032, China; 4 Institute of Neurology, Huashan Hospital, Fudan University, Shanghai 200040, China; 5 National Clinical Research Center for Aging and Medicine, Huashan Hospital, Fudan University, Shanghai 200040, China; 6 National Center for Neurological Disorders, Huashan Hospital, Fudan University, Shanghai 200040, China; 7 MOE Frontiers Center for Brain Science, Fudan University, Shanghai 200032, China

**Keywords:** Alzheimer’s disease, cognitive decline, cohort, dementia, depressive symptoms, plasma biomarkers

## Abstract

**Background:**

Depressive symptoms are closely associated with cognitive decline and risk of incident dementia, and plasma biomarkers may play a significant role in this relationship. We aimed to investigate the influence of plasma biomarkers and explore the underlying mechanisms.

**Methods:**

This study included 1,658 dementia-free community residents recruited in 2009–2011 from the Shanghai Aging Study. At baseline, we assayed plasma phosphorylated tau 217 (p-tau217) and neurofilament light chain (NfL), and assessed depressive symptoms using the Center for Epidemiologic Studies Depression scale. Cox regression models were performed to estimate the risks of incident dementia and Alzheimer’s disease (AD) during the 5-year follow-up. Parallel and serial mediation models were applied to investigate whether plasma p-tau217 and NfL mediated the relationship between depressive symptoms and cognitive decline.

**Results:**

Older adults with depressive symptoms had higher risks of dementia and AD, especially among those with higher concentrations of baseline plasma p-tau217/NfL. Sex-specific analysis revealed that depressive symptoms combined with high plasma NfL increased AD risk in men (hazard ratio, HR [95% confidence interval, CI] = 5.89 [2.01, 17.27], *p* = 0.001), whereas women with depressive symptoms and high plasma p-tau217 showed higher AD risk (HR [95%CI] = 6.07 [2.82, 13.09], *p* < 0.001). Parallel mediation analysis revealed that plasma p-tau217/NfL mediated the relationship between depressive symptoms and cognitive decline, respectively. Additionally, serial mediation analysis found p-tau217 precedes NfL within the mediating pathway (*β* = 0.403, bootstrap 95% CI: 0.347, 0.452).

**Conclusions:**

Plasma p-tau217 and NfL could individually or jointly mediate the relationship between depressive symptoms and cognitive decline.

## Introduction

Dementia poses a growing public health concern due to the increasing prevalence and the associated societal and economic impact. Approximately 10 million incident cases of dementia arise annually, and this figure is set to triple by 2050 (World Health Organization, 2019), highlighting the urgency of identifying risk factors for dementia or Alzheimer’s disease (AD) (Jia et al., 2020).

Depressive symptoms are closely associated with dementia and AD. Accumulating evidence from population-based longitudinal cohorts has suggested that depressive symptoms may contribute to cognitive decline or may be associated with an increased risk of dementia in older adults (Holmquist, Nordström, & Nordström, 2020; Wu et al., 2024; Yu, Jung, Go, Park, & Ha, 2020). However, the underlying mechanism that links the two pathologies is still uncertain.

Plasma biomarkers offer an opportunity as a cost- and time-effective approach that is minimally invasive for screening and diagnosing dementia (Abbott, 2024), including phosphorylated tau (e.g. phosphorylated tau 217 [p-tau217]), as well as neurofilament light (NfL) (Cullen et al., 2021). Elevation of NfL levels, which might be found in normal aging or neurodegenerative diseases such as dementia, can be detected in peripheral blood with a much lower concentration than in cerebrospinal fluid (CSF; Chen et al., 2024). Notably, p-tau217 has been demonstrated to be a more valuable biomarker for predicting cognitive decline than other p-tau biomarkers (Xiao et al., 2023). Although plasma p-tau217 and NfL have shown considerable potential as clinical biomarkers, the impact of non-disease-related variables remains largely unexplored (Della Monica et al., 2024). These biomarkers can exhibit interindividual differences due to demographic or comorbid factors, and intraindividual fluctuations owing to behaviors or biological changes. Interest factors encompass demographic variables, such as age and gender, in addition to behavioral elements like depressive symptoms (Almeida, Hankey, Yeap, Golledge, & Flicker, 2017), and so forth. Recent studies have found that plasma levels of p-tau217 and NfL were higher in individuals with depressive symptoms (Al-Hakeim, Al-Naqeeb, Almulla, & Maes, 2023; Schuurmans, Ghanbari, Cecil, Ikram, & Luik, 2024). However, most current research focuses on either depressive symptoms or plasma biomarkers separately in terms of their influence on cognitive decline and risk of dementia, with very few studies combining these two factors.

Based on the Shanghai Aging Study (SAS), a community-based longitudinal cohort, this study aimed to investigate the impact of plasma biomarkers on the association between depressive symptoms and cognitive decline or the risk of dementia. We also intended to explore the pathway between p-tau217 and NfL, offering insights for clinical practice and basic medical research.

## Methods and materials

### Study participants

The SAS was a community-based longitudinal cohort study that aimed to establish a prospective cohort to investigate the incidence of dementia and AD among older residents in the Jingansi community of Shanghai, China. At baseline, the SAS enrolled 3,141 registered residents aged 60 years or older in this community after excluding participants if they (a) had severe schizophrenia or mental retardation documented in their medical records, or (b) had severe vision, hearing, or speech impairments that precluded their participation in the neuropsychological assessment. Details of the design and procedure of the SAS were published elsewhere (Ding et al., 2014).

In the current study, we excluded participants from the SAS cohort who (a) were diagnosed with dementia at baseline, (b) had their blood samples collected and preserved for biomarker assessment, or (c) were lost to follow-up. The detailed flowchart of the participant selection and exclusion process is presented in Supplementary Figure S1.

This study was approved by the Medical Ethics Committee of Huashan Hospital, Fudan University, Shanghai, China. All participants and/or their legally authorized representatives provided their written informed consent.

### Participants characteristics

Demographic characteristics, including age, sex, and years of education, were collected through a face-to-face questionnaire at baseline. Depressive symptoms were assessed using the Center for Epidemiologic Studies Depression scale (CES-D) at baseline. The CES-D is a brief and structured self-report measure of depressive symptoms that has been extensively applied over the past 40 years in epidemiological research on depressive symptoms in general populations of all ages, including the older population (Cheng & Chan, 2005). Depressive symptoms were ascertained to be present if the CES-D score was ≥16 (Yuan, Chen, Zhu, Zhang, & Jin, 1998; Zhang et al., 2008).

Apolipoprotein E (APOE) genotyping was conducted by the TaqMan single-nucleotide polymorphism method (Smirnov, Morley, Shin, Spielman, & Cheung, 2009). APOE ε4 positivity was indicated by the presence of at least one ε4 allele.

### Blood biomarker assays

At baseline, overnight fasting blood samples were collected, centrifuged, and stored at −80 °C. These samples were later used for biomarker assays in August 2023 (p-tau217) and from November 2019 to January 2023 (NfL) (Xiao et al., 2023).

EDTA plasma p-tau217 and NfL were measured utilizing an ultrasensitive single-molecule array (Simoa) technology (Quanterix) on the automated Simoa HD-X platform (GBIO). The ALZ-path Simoa p-tau217 v2 (Cat No: 104371) (Chenna et al., 2023) and NfL (Cat No: 103186) (Xiao et al., 2021) assay kits were used according to the technical notes. Samples were diluted at a 1:3 ratio for the assay of p-tau217 and at a 1:4 ratio for the assays of NfL. Both calibrators and quality controls were measured in duplicate. Sample measurement was performed in a single-run mode using kits with the same lot numbers. Operators were blind to participants’ characteristics.

### Assessment of cognition and diagnosis

Each participant completed a series of neuropsychological tests designed to assess cognitive functions, including (1) Mini-Mental State Examination (MMSE) for global cognitive function; (2) Conflicting Instructions Task (Go/No Go Task) for executive function; (3) Stick Test for spatial construction function; (4) Modified Common Objects Sorting Test for language function; (5) Auditory Verbal Learning Test for memory function; (6) Modified Fuld Object Memory Evaluation for memory function; (7) Trail-making Test A&B for attention function; (8) Renminbi (official currency of China) Test for attention function, translated from the EURO test. Tests (1) to (5) and (7) were used for subjects with less than 6 years of education, while tests (1) to (4) and (6) and (8) were employed for subjects with at least 6 years of education. A multidisciplinary expert panel consisting of two neurologists, one neuropsychologist, and one neuroepidemiologist made a consensus diagnosis considering all neurological and medical examinations (Ding et al., 2014).

Dementia was diagnosed according to Diagnostic and Statistical Manual of Mental Disorders, fourth edition criteria (American Psychiatric Association, 1994), and the National Institute of Neurological and Communicative Disorders and Stroke-Alzheimer Disease and Related Disorders Association criteria (McKhann et al., 1984) was applied to diagnose AD.

### Follow-up procedure

From April 2014 to December 2016, dementia-free participants with blood samples stored at baseline underwent their first follow-up, employing the same neuropsychological tests to assess cognitive functions, and the same expert panel established a consensus diagnosis of incident dementia based on the baseline criteria. The outcomes of this study encompassed the diagnoses of dementia or AD and cognitive decline, quantified by the annual rate of MMSE score decline.

### Statistical analysis

Comparisons of baseline characteristics were tested by either Mann–Whitney *U* test (continuous data) or Pearson’s chi-square test (categorical data). Incidence rates of dementia and AD were calculated as the number of new-onset cases divided by the overall person-years of follow-up and described as ‘per 100 person-years’.

Plasma NfL was categorized into low (≤15.95 pg/mL) and high (>15.95 pg/mL) levels based on the median value of concentration. For plasma p-tau217, the derived cutoff of 0.42 pg/mL was adopted (Ashton et al., 2024). Then, participants were divided into four subgroups based on depressive symptoms and biomarker levels (Tian et al., 2020; Xiao et al., 2023): (a) high NfL/p-tau217 with depressive symptoms, (b) low NfL/p-tau217 with depressive symptoms, (c) high NfL/p-tau217 without depressive symptoms, and (d) low NfL/p-tau217 without depressive symptoms.

The cumulative incidence of dementia and AD was presented with Kaplan–Meier curves and compared using the log-rank test. Follow-up time was defined as the time from baseline to the diagnosis of dementia or to the last follow-up visit. Hazard ratios (HRs) for dementia/AD by depressive symptoms or four categorized subgroups were estimated using Cox proportional hazards regression, adjusting for age, sex, years of education, APOE ε4, hypertension, hyperlipidemia, coronary heart disease, cigarette smoking, alcohol drinking, and body mass index (BMI). Following the prior analysis, we conducted three sex-stratified Cox proportional hazards regression models. Model 1 was the univariate model. Model 2 adjusted for baseline age, years of education, and APOE ε4. Model 3 further adjusted for hypertension, hyperlipidemia, coronary heart disease, cigarette smoking, alcohol drinking, and BMI. The Schoenfeld residual-based test was used to check the proportional hazard (PH) assumption, and *p*-values of all variables were >0.05 (Grambsch & Therneau, 1994). Thus, it is reasonable to assume that the PH assumption was not violated.

We conducted a series of sensitivity analyses to examine the robustness of our findings: (1) inverse probability of censoring weighting (IPCW) analysis to minimize the influence of loss to follow-up; (2) redefining depressive symptoms with CES-D ≥ 20 to address missing historical data; (3) performing continuous analysis using generalized additive models (GAMs); (4) repeating subgroup analysis using the median of p-tau217 as the cutoff; (5) incorporating the interaction terms between sex and each biomarker (p-tau217 and NfL) into original Cox models.

To explore the pathways linking depressive symptoms to cognitive decline, the macro-program PROCESS was applied to develop parallel and serial mediation models (W. Q. Xu et al., 2021). The parallel mediation model (PROCESS Model 4.2) was employed to explore the independent mediating roles of p-tau217 and NfL in the relationship between depressive symptoms and the annual rate of decline in MMSE (Hayes, 2013). In this model, depressive symptoms were set as the predictor variable, the annual rate of MMSE decline as the outcome variable, and plasma p-tau217 and NfL as parallel mediators. A serial mediation model (PROCESS Model 6) was performed to further investigate the sequential mediating pathway among the two mediators (Hayes, 2012). Based on existing biological mechanisms whereby hyperphosphorylated tau accumulation induces neuroaxonal damage and subsequent elevation of peripheral blood NfL levels (Teunissen et al., 2022), we built a serial mediation model with the sequence ‘depressive symptoms → p-tau217 → NfL → cognitive decline’ to test if depressive symptoms affect cognitive decline through the sequential effect of p-tau217 and NfL. Before analysis, original concentrations of plasma p-tau217 and NfL were log10 transformed, and following the identification of outliers, CES-D scores of depressive symptoms and the annual MMSE decline rate (i.e. [baseline MMSE score – follow-up MMSE score]/follow-up years) were standardized. The significance of each path was evaluated using bias-corrected percentile bootstrap confidence intervals (CIs), which indicate significance when they do not include zero.

Data analyses were conducted using R version 4.4.1 with the ‘survminer’ package for cumulative risk curves, the ‘survival’ package for Cox proportional hazards models, and the ‘processR’ and ‘lavaan’ packages for parallel and serial mediation models. All *p*-values and 95% CIs were estimated using a two-tailed test, with a threshold for statistical significance at *p* < 0.05.

## Results

### Characteristics of study participants

Table 1 compares baseline and follow-up characteristics of the participants according to depressive symptoms. Among the 1,658 participants included in this study, 244 (14.7%) individuals suffered from depressive symptoms. During the median 5.3 (range 0.9–7.3) years of follow-up, the incidence rates of dementia and AD were, respectively, 2.8/100 and 2.1/100 person-years in participants with depressive symptoms, and 1.8/100 and 1.3/100 person-years in those without depressive symptoms. Participants with depressive symptoms were more likely to develop dementia (*p* = 0.022) and AD (*p* = 0.030) compared with those without depressive symptoms. We also compared baseline demographic and clinical characteristics of enrolled participants and non-enrolled participants (Supplementary Table S4). The results indicated no statistically significant differences in the key variables involved in the study.

**Table 1.** Baseline and follow-up characteristics between participants with and without depressive symptoms

	Without depressive symptoms	With depressive symptoms	*p*
	*N* = 1,414	*N* = 244	
**Baseline**			
Age, years, mean ± SD	71.4 ± 7.5	72.1 ± 7.1	0.129
Sex, male, *n* (%)	661 (46.7)	98 (40.2)	0.066
APOE ε4, positive, *n* (%)	230 (16.3)	42 (17.2)	0.783
Education, years, mean ± SD	11.9 ± 4.0	11.9 ± 4.1	0.834
MMSE, mean ± SD	28.2 ± 2.0	28.0 ± 2.2	0.050
Cigarette smoking, *n* (%)	294 (20.8)	40 (16.4)	0.135
Alcohol drinking, *n* (%)	131 (9.3)	18 (7.4)	0.406
BMI, mean ± SD	24.7 ± 3.5	24.1 ± 3.5	0.021
Hypertension, *n* (%)	756 (53.5)	139 (57.0)	0.345
Hyperlipidemia, *n* (%)	505 (35.7)	104 (42.6)	0.046
Coronary heart disease, *n* (%)	152 (10.7)	42 (17.2)	0.005
NfL, pg/mL, mean ± SD	18.8 ± 12.9	20.0 ± 12.4	0.260
p-tau217, pg/mL, mean ± SD	0.4 ± 0.3	0.4 ± 0.4	0.464
**Follow-up**			
Follow-up years, median (range)	5.3 (0.9, 7.3)	5.2 (1.0, 7.1)	0.783
MMSE, mean ± SD	26.7 ± 4.1	26.4 ± 3.9	0.394
Incident dementia, *n* (rate/100 person-years)	132 (1.8/100)	35 (2.8/100)	0.022
Incident AD, *n* (rate/100 person-years)	97 (1.3/100)	27 (2.1/100)	0.030

*Note*: Continuous variables were compared with the Mann–Whitney *U* test, and categorical variables were compared with Pearson’s chi-squared test. Abbreviations: AD, Alzheimer’s disease; APOE, apolipoprotein E; BMI, body mass index; MMSE, Mini-Mental State Examination; NfL, neurofilament light chain; p-tau217, phosphorylated tau 217; SD, standard deviation.

### Associations of depressive symptoms with incident dementia and AD

**Figure 1.**
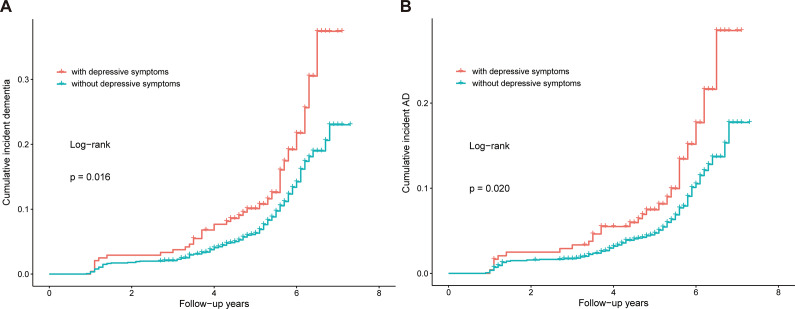
Kaplan–Meier cumulative risk curves for dementia (A) and AD (B) by baseline depressive symptoms status based on the CES-D assessment. *Note*: With depressive symptoms (CES-D score ≥ 16); without depressive symptoms *n* (CES-D score < 16). Abbreviations: AD, Alzheimer’s disease.

As shown in Figure 1, the cumulative incidence rate of dementia and AD increased faster among depressed participants than among those without depressive symptoms (log-rank test, dementia: *p* = 0.016; AD: *p* = 0.020). Adjusting for age, sex, education, cigarette smoking, alcohol drinking, BMI, hypertension, hyperlipidemia, coronary heart disease, and APOE ε4, the Cox regression model showed that participants with depressive symptoms had significantly higher risks of incident dementia (HR [95% CI] = 1.59 [1.09, 2.33], *p* = 0.016) and AD (HR [95% CI] = 1.70 [1.10, 2.62], *p* = 0.017) than those without (Supplementary Table S1).

### Incident dementia and AD across subgroups by depressive symptoms and plasma biomarkers

**Figure 2.**
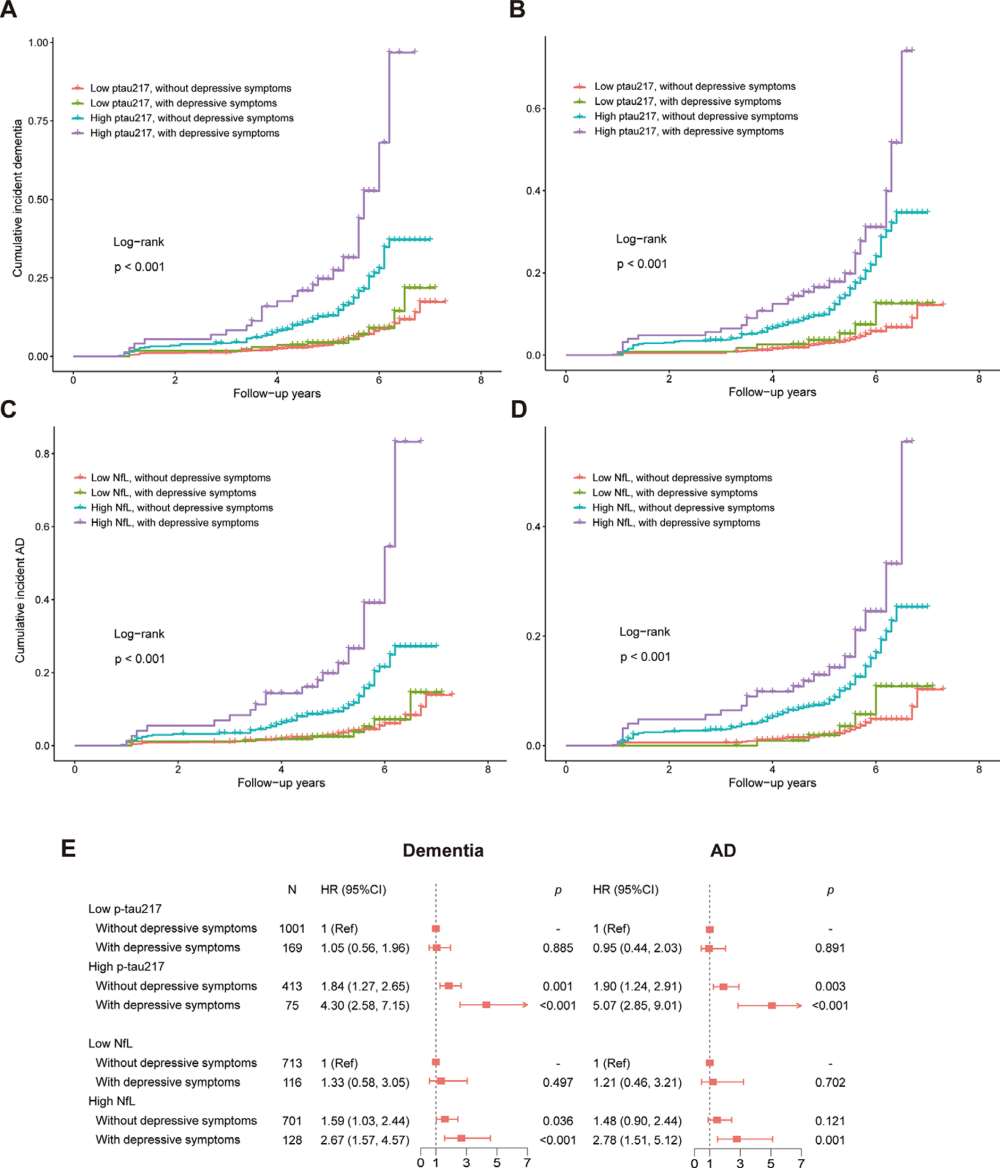
Subgroup analyses of depressive symptoms and p-tau217/NfL on incident dementia and AD. (A–D) Comparisons of four subgroups combined different levels of p-tau217/NfL and depressive symptoms by the Kaplan–Meier estimations, log-rank tests. (E) Forest plots for hazard ratios of incident dementia and AD in four subgroups, derived from Cox regression models with adjustments for age, sex, education years, APOE ε4, hypertension, hyperlipidemia, coronary heart disease, cigarette smoking, alcohol drinking, and BMI at baseline. The low p-tau217, ≤0.42 pg/mL; high p-tau217, >0.42 pg/mL. The low NfL, ≤15.95 pg/mL; high p-tau217, >15.95 pg/mL. With depressive symptoms, score of CES-D ≥ 16; without depressive symptoms, score of CES-D < 16. Abbreviations: AD, Alzheimer’s disease; APOE, apolipoprotein E; CI, confidence interval; HR, hazard ratio; NfL, neurofilament light chain; p-tau217, phosphorylated tau 217.

As shown in Figure 2A–D, there were significant differences of cumulative incident dementia/AD rates among the four subgroups stratified by plasma biomarker levels and depressive symptoms (log-rank test, *p* < 0.001). After adjusting for age, sex, education, APOE ε4, hypertension, hyperlipidemia, coronary heart disease, cigarette smoking, alcohol drinking, and BMI, Cox regression models revealed that participants with depressive symptoms and high biomarker levels demonstrated higher risk of dementia (HR_p-tau217_ [95% CI] = 4.30 [2.58, 7.15], *p* < 0.001; HR_NfL_ [95% CI] = 2.67 [1.57, 4.57], *p* < 0.001; Figure 2E) and AD (HR_p-tau217_ [95% CI] = 5.07 [2.85, 9.01], *p* < 0.001; HR_NfL_ [95% CI] = 2.78 [1.51, 5.12], *p* < 0.001; Figure 2E).

### Sex differences in the risks of dementia and AD across subgroups

Sex-specific Cox models revealed differences in how plasma biomarkers and depressive symptoms jointly influence dementia/AD risk. In men, elevated dementia/AD risk was observed among those with both high plasma NfL levels (>15.95 pg/mL) and depressive symptoms (Model 3 adjusted HR [95% CI] = 4.17 [1.65, 10.53], *p* = 0.003 for dementia; Model 3 adjusted HR [95% CI] = 5.89 [2.01, 17.27], *p* = 0.001 for AD; Table 2). In women, higher risk of dementia/AD was observed in the subgroup with high plasma p-tau217 levels (>0.42 pg/mL) and depressive symptoms (Model 3 adjusted HR [95% CI] = 5.48 [2.89, 10.40], *p* < 0.001 for dementia; Model 3 adjusted HR [95% CI] = 6.07 [2.82, 13.09], *p* < 0.001 for AD; Table 2).

**Table 2.** Sex-specific hazard ratios for incident dementia and AD across four subgroups

		Men (*N* = 759)					
		Model 1		Model 2		Model 3	
	*N*	HR (95% CI) for dementia	*p*	HR (95% CI) for dementia	*p*	HR (95% CI) for dementia	*p*
Low p-tau217							
Without depressive symptoms	445	1 (Ref)	–	1 (Ref)	–	1 (Ref)	–
With depressive symptoms	60	1.76 (0.67, 4.65)	0.255	2.39 (0.90, 6.37)	0.082	2.50 (0.91, 6.82)	0.074
High p-tau217							
Without depressive symptoms	216	2.23 (1.26, 3.93)	0.006	1.22 (0.67, 2.23)	0.514	1.18 (0.64, 2.19)	0.593
With depressive symptoms	38	5.67 (2.60, 12.35)	<0.001	3.23 (1.39, 7.52)	0.006	2.76 (1.14, 6.70)	0.024
Low NfL							
Without depressive symptoms	311	1 (Ref)	–	1 (Ref)	–	1 (Ref)	–
With depressive symptoms	39	1.64 (0.36, 7.41)	0.522	0.75 (0.16, 3.55)	0.718	0.63 (0.13, 3.08)	0.57
High NfL							
Without depressive symptoms	350	3.34 (1.70, 6.56)	<0.001	1.09 (0.52, 2.30)	0.82	0.97 (0.45, 2.08)	0.94
With depressive symptoms	59	7.30 (3.20, 16.65)	<0.001	4.78 (1.96, 11.64)	<0.001	4.17 (1.65, 10.53)	0.003
	N	HR (95%CI) for AD	*p*	HR (95%CI) for AD	*p*	HR (95%CI) for AD	*p*
Low p-tau217							
Without depressive symptoms	445	1 (Ref)	–	1 (Ref)	–	1 (Ref)	–
With depressive symptoms	60	1.82 (0.61, 5.43)	0.28	2.61 (0.87, 7.86)	0.088	2.52 (0.81, 7.81)	0.109
High p-tau217							
Without depressive symptoms	216	2.00 (1.03, 3.88)	0.041	1.03 (0.51, 2.08)	0.934	0.95 (0.46, 1.96)	0.89
With depressive symptoms	38	7.34 (3.26, 16.53)	<0.001	4.02 (1.65, 9.78)	0.001	3.48 (1.35, 8.95)	0.01
Low NfL							
Without depressive symptoms	311	1 (Ref)	–	1 (Ref)	–	1 (Ref)	–
With depressive symptoms	39	2.59 (0.54, 12.53)	0.236	1.07 (0.21, 5.49)	0.938	0.83 (0.16, 4.44)	0.832
High NfL							
Without depressive symptoms	350	3.98 (1.73, 9.14)	0.001	1.17 (0.47, 2.90)	0.731	1.02 (0.40, 2.58)	0.968
With depressive symptoms	59	10.55 (4.05, 27.46)	<0.001	6.70 (2.40, 18.72)	<0.001	5.89 (2.01, 17.27)	0.001

		Women (*N* = 899)					
		Model 1		Model 2		Model 3	
	*N*	HR (95% CI) for dementia	*p*	HR (95% CI) for dementia	*p*	HR (95% CI) for dementia	*p*
Low p-tau217							
Without depressive symptoms	556	1 (Ref)	–	1 (Ref)	–	1 (Ref)	–
With depressive symptoms	109	0.92 (0.41, 2.10)	0.849	0.80 (0.35, 1.79)	0.581	0.83 (0.37, 1.88)	0.661
High p-tau217							
Without depressive symptoms	197	4.41 (2.86, 6.80)	<0.001	2.34 (1.48, 3.71)	<0.001	2.46 (1.55, 3.92)	<0.001
With depressive symptoms	37	8.33 (4.48, 15.50)	<0.001	4.96 (2.64, 9.34)	<0.001	5.48 (2.89, 10.40)	<0.001
Low NfL							
Without depressive symptoms	402	1 (Ref)	–	1 (Ref)	–	1 (Ref)	–
With depressive symptoms	77	1.43 (0.53, 3.86)	0.476	1.63 (0.60, 4.39)	0.339	1.70 (0.63, 4.63)	0.295
High NfL							
Without depressive symptoms	351	4.79 (2.84, 8.08)	<0.001	2.02 (1.19, 3.44)	0.009	2.25 (1.31, 3.88)	0.003
With depressive symptoms	69	5.76 (2.93, 11.31)	<0.001	2.25 (1.14, 4.47)	0.02	2.63 (1.31, 5.28)	0.006
	N	HR (95%CI) for AD	*p*	HR (95%CI) for AD	*p*	HR (95%CI) for AD	*p*
Low p-tau217							
Without depressive symptoms	556	1 (Ref)	–	1 (Ref)	–	1 (Ref)	–
With depressive symptoms	109	0.76 (0.26, 2.18)	0.608	0.66 (0.23, 1.90)	0.439	0.66 (0.23, 1.91)	0.444
High p-tau217							
Without depressive symptoms	197	4.90 (2.95, 8.16)	<0.001	2.58 (1.50, 4.44)	<0.001	2.87 (1.66, 4.96)	<0.001
With depressive symptoms	37	8.64 (4.13, 18.09)	<0.001	5.36 (2.52, 11.39)	<0.001	6.07 (2.82, 13.09)	<0.001
Low NfL							
Without depressive symptoms	402	1 (Ref)	–	1 (Ref)	–	1 (Ref)	–
With depressive symptoms	77	1.03 (0.30, 3.56)	0.961	1.22 (0.35, 4.24)	0.754	1.23 (0.35, 4.27)	0.747
High NfL							
Without depressive symptoms	351	4.09 (2.29, 7.33)	<0.001	1.68 (0.93, 3.04)	0.088	1.78 (0.97, 3.26)	0.061
With depressive symptoms	69	4.75 (2.18, 10.37)	<0.001	1.86 (0.84, 4.11)	0.124	2.02 (0.90, 4.52)	0.089

*Note*: Model 1: Univariate Cox regression model; Model 2: Multivariate Cox regression model, adjusted for baseline age, years of education, and APOE ε4; Model 3: Multivariate Cox regression model, adjusted for baseline age, years of education, APOE ε4, hypertension, hyperlipidemia, coronary heart disease, cigarette smoking, alcohol drinking, and BMI.

The low p-tau217, ≤0.42 pg/mL; high p-tau217, >0.42 pg/mL. The low NfL, ≤15.95 pg/mL; high p-tau217, >15.95 pg/mL. With depressive symptoms, score of CES-D ≥ 16; without depressive symptoms, score of CES-D < 16.

Abbreviations: AD, Alzheimer’s disease; CI, confidence interval; HR, hazard ratio; NfL, neurofilament light chain; p-tau217, phosphorylated tau 217.

### Sensitivity analysis

In sensitivity analysis, the risks of incident dementia and AD by depressive symptoms and plasma biomarkers were consistent across several conditions: (1) using IPCW models (Supplementary Table S5); (2) using Cox-GAMs (Supplementary Figures S2 and S3); (3) using the median of p-tau217 (0.32 pg/mL) as the cutoff value (Supplementary Table S11); and (4) incorporating the interaction terms between sex and plasma biomarker (Supplementary Table S12).

Similar results were also observed in analyses where depressive symptoms were defined as CES-D ≥ 20, including comparisons of participants’ characteristics, multivariable, subgroup, and sex-specific Cox regression analyses (Supplementary Tables S6–S10).

### Parallel mediation model

**Figure 3.**
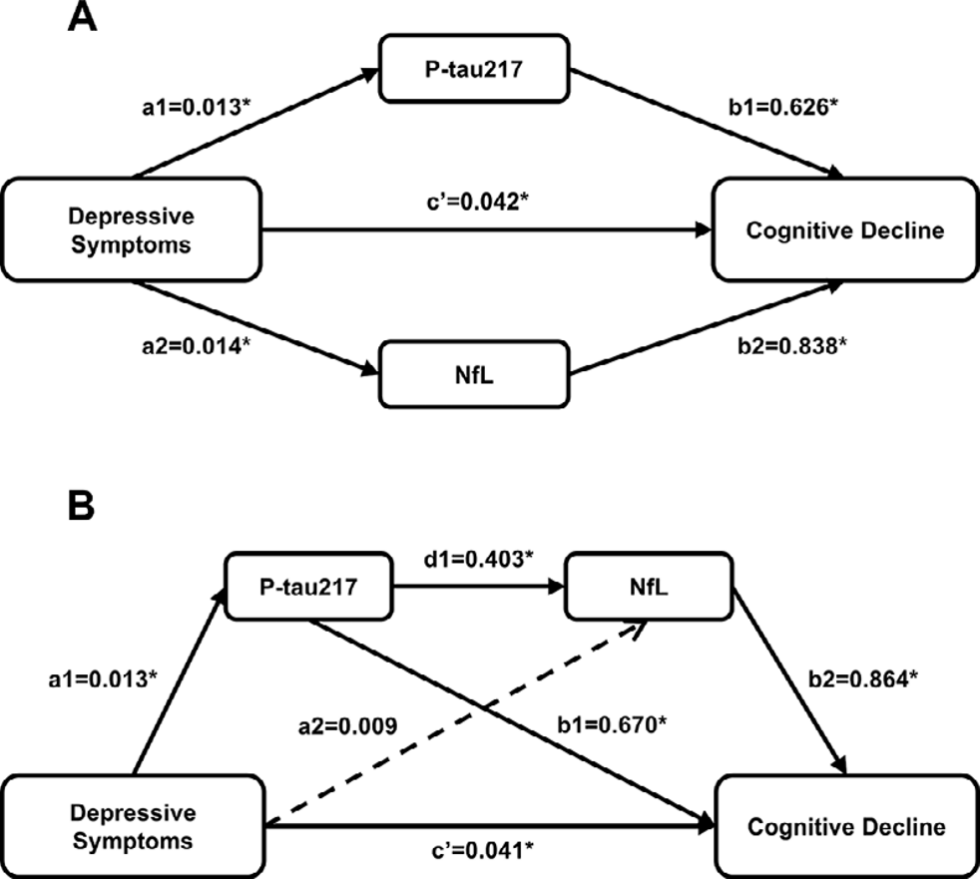
Parallel and serial mediation models. (A) Parallel mediation model included the direct and indirect effects of depressive symptoms on cognitive decline through plasma p-tau217 and NfL. (B) Serial mediation model included pathway depressive symptoms → p-tau217 → NfL → cognitive decline. All presented effects are unstandardized. *Significant at the 0.05 level (two-tailed). Continuous lines denoted significant regression. Abbreviations: NfL, neurofilament light chain; p-tau217, phosphorylated tau 217.

As shown in Figure 3A and Supplementary Table S2, the parallel mediation model demonstrated that plasma p-tau217/NfL could respectively mediate the relationship between depressive symptoms and cognitive decline (a1*b1 = 0.008, bootstrap 95% CI: 0.001, 0.022; a2*b2 = 0.012, bootstrap 95% CI: 0.003, 0.026). The direct effect of depressive symptoms on cognitive decline was also significant (*c*’ = 0.042, bootstrap 95% CI: 0.006, 0.088). The mediating effect sizes of p-tau217 and NfL accounted for 12.90% and 19.35% of the total effect, respectively, indicating that the two plasma biomarkers partially mediated the association between depressive symptoms and cognitive decline. Detailed information on the total effect, direct effect, indirect effect, and corresponding 95% CIs of the parallel mediation model was presented in Supplemental Table S2.

### Serial mediation model

The serial mediation model was constructed to explore the sequential mediating role of plasma p-tau217 and NfL according to their pathophysiological sequence, with the coefficients and significance of each path presented in Figure 3B. The results showed that the direct path from depressive symptoms to cognitive decline remained significant (*c*’ = 0.041, 95% CI: 0.002, 0.084), consistent with the parallel mediation model. For indirect paths, the serial path ‘depressive symptoms → p-tau217 → NfL → cognitive decline’ was statistically significant, indicating that depressive symptoms could induce cognitive decline through the sequential effects of p-tau217 and NfL. In contrast to the parallel mediation model, the single mediating path of NfL was not significant (a2*b2 = 0.007, bootstrap 95% CI: −0.003, 0.017), while plasma p-tau217 was significantly positively associated with NfL (d1 = 0.403, bootstrap 95% CI: 0.347, 0.452), confirming the hypothetical pathophysiological sequence that p-tau217 accumulation precedes NfL elevation. The total effect, direct effect, and specific indirect effects of the serial mediation model were detailed in Supplemental Table S3.

## Discussion

This study demonstrated that older adults with depressive symptoms exhibited higher risks of dementia and AD, especially among those with elevated baseline plasma p-tau217 or NfL concentration. In the sex-stratified analyses, we found that depressive symptoms combined with high plasma NfL levels (>15.95 pg/mL) increased dementia/AD risk in men, whereas for women, a higher risk of dementia/AD was observed in those with high plasma p-tau217 levels (>0.42 pg/mL) and depressive symptoms. Parallel mediation analysis revealed that plasma p-tau217/NfL mediated the relationship between depressive symptoms and cognitive decline, respectively. Additionally, serial mediation analysis found a significant serial chain of depressive symptoms → p-tau217 → NfL → cognitive decline.

The 2024 Lancet Commission report on dementia highlighted that depressive symptoms contributed to the development of dementia in later life (age > 65 years), accounting for a weighted population-attributable fraction of 3% (Livingston et al., 2024). Our results confirmed that depressive symptoms were associated with higher risks of dementia and AD, which was consistent with previous population studies. A 14-year longitudinal study of 4,922 cognitively healthy men aged 71–89 years indicated that older men with depressive symptoms were at increased risk of developing dementia (Almeida et al., 2017). A meta-analysis of 23 prospective population studies revealed that late-life depression significantly increased the risk of future AD incidence, suggesting its considerable influence as a risk factor after adjustment for multiple confounders (Carles et al., 2020). Previous studies have also identified depressive symptoms as a risk factor for cognitive decline. The Survey of Health, Aging and Retirement in Europe, with a sample size of 14,231 individuals aged 60 years and older, and the China Health and Retirement Longitudinal Study, with 7,335 participants aged 45 years and above, both indicated an association between depressive symptoms and the prevalence of cognitive decline (Han et al., 2021; Yang et al., 2020).

Few population studies considered the roles of plasma p-tau217 and NfL on the association between depressive symptoms and cognitive decline or dementia onset. In individuals diagnosed with AD, there were observed associations between depressive symptoms and smaller hippocampal volume, higher tau accumulation, and amyloid metabolism (Chi, Yu, Tan, & Tan, 2014), suggesting that depressive symptoms might be associated with underlying brain changes. Plasma biomarkers are promising in the diagnosis and prognosis of AD, including amyloid-beta (Aβ40 and Aβ42), phosphorylated tau (p-tau181 and p-tau217), glial fibrillary acidic protein, and NfL (Blennow et al., 2023; Teunissen et al., 2022). In cognitively unimpaired older adults, depressive symptoms were linked to the presence of Aβ (A), tau (T), and neurodegeneration (N), collectively termed the ATN system (Rubin-Norowitz, Lipton, Petersen, & Ezzati, 2022), indicative of AD pathology. The majority of earlier studies focused more on biomarkers derived from positron emission tomography (PET) and CSF than on plasma biomarkers (Twait et al., 2024). Using data from the Alzheimer’s Disease Neuroimaging Initiative, a longitudinal cohort of older individuals with an emphasis on the collection of biomarkers accessed via CSF (Rubin-Norowitz et al., 2022) or PET (Touron et al., 2022), the association between depressive symptoms and cognitive function was highly influenced by the AT(N) biomarker profile. Xu et al. (2021) found that (1) depressive symptoms could significantly elevate the risk of cognitive decline and AD; (2) depressive symptoms predicted greater CSF amyloid burden; and (3) amyloid pathology might partially mediate the impact of depressive symptoms on cognitive impairments and AD risk. Synthesizing prior studies, it was evident that biomarkers found in CSF or detected through PET scans gained more concentration, particularly Aβ or its combination with other biomarkers. Meanwhile, studies that concurrently investigate plasma p-tau217 and NfL in relation to cognitive decline or the risk of dementia were relatively scarce.

The present study demonstrated that the serial mediation effect of p-tau217 and NfL in sequence and the separate mediation effect of them in the relationship between depressive symptoms and cognitive decline were both statistically significant. Pathological and basic research have provided clues to explain the underlying mechanisms. Hyperphosphorylation of tau, along with other posttranslational modifications, predisposes it to aggregate more readily, potentially resulting in the formation of fibrillar tau deposits in neurofibrillary tangles, the main intraneuronal lesions in AD (Aragão Gomes et al., 2021). The lesions primarily accumulate in the transentorhinal and entorhinal regions, which include the hippocampus, an area where p-tau217 first appears and is particularly vulnerable to age-related changes. Besides, increased CSF concentrations of NfL correlate with accelerated cognitive decline, white matter lesions, and increased brain atrophy in participants with AD dementia, indicating that degeneration of large-caliber axons is an important element of disease progression in AD (Salwierz et al., 2024; Zetterberg et al., 2016). Remarkably, our serial mediation model, incorporating both p-tau217 and NfL, revealed a statistically significant path between them; however, the mediating effect of NfL alone was no longer evident. Recent review demonstrated that hyperphosphorylated tau induced neurofibrillary tangle formation and neuronal cytoskeletal disruption, which in turn triggered NfL release into the CSF and peripheral blood (Teunissen et al., 2022). Longitudinal study has also shown that elevated p-tau217 levels can be detected years before the onset of cognitive impairment, while NfL elevation typically occurs later, coinciding with the progression of neurodegeneration (Mattsson-Carlgren et al., 2020). Furthermore, a multicenter, nested case–control study with cognitively normal participants enrolled in the China Cognition and Aging Study found the time courses of CSF biomarkers, in which tau appeared ~1–2 years before NfL (*Jia et al., 2024*). A clinically validated quantitative pharmacology model, supported by in vitro neuronal experimental data, identified neuronal homeostasis disruption and cytoskeletal degradation caused by hyperphosphorylated tau as key mechanisms driving NfL release (Maliukova & Karelina, 2025).

Furthermore, the present study found a synergistic effect between depressive symptoms and high levels of plasma biomarkers on the risks of dementia and AD, with results also indicating sex differences. Both Almeida et al. and Heser et al. confirmed that older men with depressive symptoms were associated with increased risk of incident dementia (Almeida et al., 2017; Heser et al., 2020). However, the results from the Three-City Study demonstrated that women with depressive symptoms confirmed by CES-D had higher dementia risk (Norton et al., 2019). These contradictory findings might be attributed to the heterogeneity of the study populations, the diversity in methodological approaches, and the absence of baseline pathological assessments. Although sex differences in depressive symptoms and cognitive function were frequently documented (Gong et al., 2023), our understanding of the neurobiological processes that account for these differences was limited. Previous studies showed that estrogen, particularly 17β-estradiol, can reduce tau hyperphosphorylation and modulate Aβ metabolism, while postmenopausal estrogen decline increased the risks of diseases associated with AD pathology (Mohammed et al., 2025). For men, although testosterone was generally considered to have neuroprotective effects, age-related decline in testosterone levels could disrupt neurotransmitter balance (e.g. serotonin) (Rubin-Norowitz et al., 2022), which may exacerbate neuroaxonal damage and synergize with depressive symptoms to increase dementia risk. Our recent work also reported that testosterone and NfL exerted a joint effect on cognitive decline in older men (Tang et al., 2024). Furthermore, men had more unhealthy behaviors (e.g. substance abuse) compared to women, which can induce neuroaxonal injury and elevated plasma NfL (Bavato et al., 2024).

Our findings confirmed that plasma biomarkers exert a significant role in the association between depressive symptoms and cognitive decline. Integrating these plasma biomarkers into community screening practices could enable early identification of high-risk individuals with cognitive impairment, which is critical for initiating timely and targeted interventions. To optimize cost-effectiveness, plasma biomarker testing can be targeted at individuals positive for preliminary depression screening rather than the general population. Although plasma biomarker assays cost more than questionnaires, they remain markedly more accessible and less invasive than CSF testing and PET scanning, and early intervention guided by accurate screening can reduce long-term medical and nursing expenditures related to neurodegenerative diseases.

Strengths of the current study included the prospective study design, a relatively large and representative population sample, and consensus diagnosis of dementia at baseline and follow-up. Besides, the SAS detected biomarkers using fasting blood samples, which could reduce the impact of external factors on biomarker measurements. Lastly, the current study integrated demographic, behavioral, and laboratory plasma indicators, providing a comprehensive view of cognitive decline and dementia risk, something rarely achieved in earlier studies. There are also some limitations in this study. First, the outcomes of this study encompassed cognitive decline, quantified by the rate of MMSE decline, and the diagnosis of dementia or AD, which relied on clinical diagnostic criteria but lacked pathological confirmation. In a large community-based population study, obtaining pathological confirmation that requires invasive procedures is usually not feasible due to ethical considerations and participant compliance issues. Second, although we made adjustments for some cofounders, including sex, age, education years, APOE ε4, hypertension, hyperlipidemia, coronary heart disease, cigarette smoking, alcohol drinking, and BMI, there could still be other confounding variables that were not taken into account in our study. For example, the failure to incorporate inflammatory markers and the use of antidepressants could affect the role played by depressive symptoms (Bocharova et al., 2025; Wang et al., 2023). Third, depressive symptoms were assessed over the past week using self-reported data at baseline, thereby failing to capture their long-term history and longitudinal changes. Also, the CES-D scale could be used to assess the current depressive status, but it was not intended for diagnosing depressive disorders. Fourth, since there is no universally accepted cutoff value, we categorized NfL into high and low concentration levels using the statistical median value, an approach specific to the dementia-free participants in our current cohort. Finally, the recruitment of participants for this study was confined to a metropolis in China, which implies that the generalizability of our results to other older adult populations might be limited. Therefore, subsequent studies should involve large, ethnically diverse samples with long follow-ups to verify our findings.

In conclusion, plasma p-tau217 and NfL could individually or jointly mediate the relationship between depressive symptoms and cognitive decline. Depressive symptoms combined with high levels of plasma p-tau217 and NfL may result in a higher risk of dementia. Additionally, the exploration of pathway mechanisms involving depressive symptoms, plasma biomarkers, and cognitive decline may serve as a foundation for clinical basic research in the future.

## Supporting information

10.1017/S0033291725103115.sm001 Xia et al. supplementary material Xia et al. supplementary material

## Supplementary material

The supplementary material for this article can be found at http://doi.org/10.1017/S0033291725103115.
