# Supplementary material for: Plasma phosphorylated tau 217 and neurofilament light chain on the association between depressive symptoms and cognitive decline: The Shanghai Aging Study
Source: Psychol Med. 2026 Jan 20;56:e25. doi: 10.1017/S0033291725103115 (PMC12885335; doi:10.1017/S0033291725103115)
Supplement: Xia et al. supplementary material [file S0033291725103115sup001.docx]

Supplementary Material – Contents

[**Table S1.** Hazard ratios for incident dementia or AD with depressive symptoms and covariates. 2](#_Toc216876803)

[**Table S2.** Parallel mediating effects of plasma p-tau217 and NfL between depressive symptoms and cognitive decline. 3](#_Toc216876804)

[**Table S3.** Serial mediating effects of plasma p-tau217 and NfL between depressive symptoms and cognitive decline. 4](#_Toc216876805)

[**Table S4.** Baseline demographic and clinical characteristics characteristics between participants enrolled and not-enrolled. 5](#_Toc216876806)

[**Table S5.** IPCW-adjusted hazard ratios for incident dementia and AD across subgroups. 6](#_Toc216876807)

[**Table S6.** Baseline and follow-up characteristics of participants by depressive symptoms (CES-D ≥ 20). 7](#_Toc216876808)

[**Table S7.** Hazard ratios for incident dementia or AD with depressive symptoms (CES-D ≥ 20) and covariates. 9](#_Toc216876809)

[**Table S8.** Hazard ratios for incident dementia and AD across subgroups in all participants. 10](#_Toc216876810)

[**Table S9.** Hazard ratios for incident dementia and AD across subgroups in men. 11](#_Toc216876811)

[**Table S10.** Hazard ratios for incident dementia and AD across subgroups in women. 12](#_Toc216876812)

[**Table S11.** Overall and sex-specific hazard ratios for incident dementia and AD across subgroups using the median of p-tau217 (0.32pg/mL) as the cutoff. 13](#_Toc216876813)

[**Table S12.** Hazard ratios of interaction terms for incident dementia and AD. 15](#_Toc216876814)

[**Figure S1.** Flow chart of study participants. 16](#_Toc216876815)

[**Figure S2.** Predicted dose-response surface plots for the joint effects of baseline CES-D scores and p-tau217/NfL on dementia incidence. 17](#_Toc216876816)

[**Figure S3.** Predicted dose-response surface plots for the joint effects of baseline CES-D scores and p-tau217/NfL on AD incidence. 17](#_Toc216876817)

**Table S1.** Hazard ratios for incident dementia or AD with depressive symptoms and covariates.

|  | Dementia | |  | AD | |
| --- | --- | --- | --- | --- | --- |
|  | HR (95%CI) | *p* |  | HR (95%CI) | *p* |
| Depressive symptoms | 1.59 (1.09, 2.33) | 0.016 |  | 1.70 (1.10, 2.62) | 0.017 |
| Age | 1.16 (1.13, 1.19) | <0.001 |  | 1.16 (1.13, 1.20) | <0.001 |
| Female | 1.05 (0.69, 1.58) | 0.827 |  | 0.81 (0.51, 1.31) | 0.395 |
| Education years | 0.91 (0.88, 0.94) | <0.001 |  | 0.89 (0.86, 0.92) | <0.001 |
| Cigarette smoking | 1.12 (0.68, 1.86) | 0.649 |  | 0.97 (0.54, 1.74) | 0.917 |
| Alcohol drinking | 0.64 (0.31, 1.35) | 0.243 |  | 0.51 (0.20, 1.30) | 0.159 |
| BMI | 0.97 (0.93, 1.02) | 0.238 |  | 0.97 (0.91, 1.02) | 0.222 |
| Hypertension | 1.35 (0.97, 1.89) | 0.076 |  | 1.10 (0.75, 1.60) | 0.638 |
| Hyperlipidemia | 0.74 (0.52, 1.05) | 0.092 |  | 0.76 (0.50, 1.15) | 0.191 |
| Coronary heart disease | 1.15 (0.77, 1.73) | 0.486 |  | 0.99 (0.60, 1.63) | 0.971 |
| APOE ε4 | 1.34 (0.91, 1.97) | 0.138 |  | 1.51 (0.99, 2.32) | 0.058 |

Note. Multivariate Cox regression model, adjusted for age, sex, years of education, cigarette smoking, alcohol drinking, BMI, hypertension, hyperlipidemia, coronary heart disease and APOE ε4.

Abbreviations: HR, hazard ratio; CI, confidence interval; BMI, body mass index; APOE, apolipoprotein E; AD, Alzheimer’s disease.

**Table S2.** Parallel mediating effects of plasma p-tau217 and NfL between depressive symptoms and cognitive decline.

|  | Effect value | Bootstrap SE | Bootstrap 95%CI | Effect percentage |
| --- | --- | --- | --- | --- |
| **Mediator 1: p-tau217** |  |  |  |  |
| a1 | 0.013 | 0.005 | [0.001, 0.024] | - |
| b1 | 0.626 | 0.185 | [0.305, 1.028] | - |
| a1*b1 | 0.008 | 0.005 | [0.001, 0.022] | 12.90% |
| **Mediator 2: NfL** |  |  |  |  |
| a2 | 0.014 | 0.006 | [0.003, 0.027] | - |
| b2 | 0.838 | 0.207 | [0.448, 1.325] | - |
| a2*b2 | 0.012 | 0.006 | [0.003, 0.026] | 19.35% |
| **Direct effect** |  |  |  |  |
| c' | 0.042 | 0.021 | [0.006, 0.088] | 67.74% |
| **Total effect** |  |  |  |  |
| c (a1*b1+a2*b2+c’) | 0.062 | 0.022 | [0.021, 0.113] | - |

Abbreviations: SE, Standard error; CI, confidence interval; p-tau217, phosphorylated tau 217; NfL, neurofilament light chain.

**Table S3.** Serial mediating effects of plasma p-tau217 and NfL between depressive symptoms and cognitive decline.

|  | Effect value | Bootstrap SE | Bootstrap 95%CI | Effect percentage |
| --- | --- | --- | --- | --- |
| **Indirect effect** |  |  |  |  |
| a1 | 0.013 | 0.006 | [0.003, 0.026] | - |
| a2 | 0.009 | 0.005 | [-0.003, 0.018] | - |
| b1 | 0.670 | 0.162 | [0.356, 1.031] | - |
| b2 | 0.864 | 0.163 | [0.543, 1.206] | - |
| d1 | 0.403 | 0.026 | [0.347, 0.452] | - |
| a1*b1 | 0.009 | 0.005 | [0.002, 0.023] | 14.52% |
| a2*b2 | 0.007 | 0.005 | [-0.003, 0.017] | 11.29% |
| a1*d1*b2 | 0.005 | 0.002 | [0.001, 0.010] | 8.06% |
| **Direct effect** |  |  |  |  |
| c' | 0.041 | 0.021 | [0.002, 0.084] | 66.13% |
| **Total effect** |  |  |  |  |
| c (a1*b1+a2*b2+a1*d1*b2+c’) | 0.062 | 0.022 | [0.021, 0.113] | - |

Abbreviations: SE, Standard error; CI, confidence interval; p-tau217, phosphorylated tau 217; NfL, neurofilament light chain.

**Table S4.** Baseline demographic and clinical characteristics characteristics between participants enrolled and non-enrolled.

|  | Non-enrolled | Enrolled | *p* |
| --- | --- | --- | --- |
|  | N=1483 | N=1658 |  |
| Age, years, Mean ± SD | 71.3 ± 8.3 | 71.5 ± 7.4 | 0.064 |
| Sex, male, n (%) | 666 (44.9) | 759 (45.8) | 0.651 |
| APOE ε4, positive, n (%) | 259 (17.5) | 270 (16.3) | 0.404 |
| Education, years, Mean ± SD | 11.8 ± 4.2 | 11.9 ± 4.0 | 0.542 |
| Hypertension, n (%) | 785 (52.9) | 895 (54.0) | 0.581 |
| Hyperlipidemia, n (%) | 512 (34.5) | 609 (36.7) | 0.211 |
| Coronary heart disease, n (%) | 175 (11.8) | 193 (11.6) | 0.933 |
| Alcohol drinking, n (%) | 121 (8.2) | 149 (9.0) | 0.446 |
| Cigarette smoking, n (%) | 299 (20.2) | 333 (20.1) | 0.992 |
| BMI, Mean ± SD | 24.3 ± 3.4 | 24.6 ± 3.5 | 0.016 |
| CES-D, Mean ± SD | 8.3 ± 8.0 | 7.9 ± 7.7 | 0.211 |

Note. Continuous variables were compared with the Mann-Whitney U test, and categorical variables were compared with Pearson’s chi-squared test.

Abbreviations: SD, standard deviation; APOE, apolipoprotein E; BMI, body mass index; CES-D, Center for Epidemiologic Studies Depression scale.

Sensitivity analysis: using IPCW model

**Table S5.** IPCW-adjusted hazard ratios for incident dementia and AD across subgroups.

|  | Dementia | |  | AD | |
| --- | --- | --- | --- | --- | --- |
|  | HR (95%CI) | *p* |  | HR (95%CI) | *p* |
| Low p-tau217 |  |  |  |  |  |
| Without depressive symptoms | 1 (Ref) | - |  | 1 (Ref) | - |
| With depressive symptoms | 1.18 (0.53, 2.61) | 0.681 |  | 1.05 (0.39, 2.82) | 0.919 |
| High p-tau217 |  |  |  |  |  |
| Without depressive symptoms | 1.82 (1.11, 3.00) | 0.017 |  | 1.92 (1.03, 3.56) | 0.039 |
| With depressive symptoms | 4.66 (2.52, 8.63) | <0.001 |  | 5.34 (2.74, 10.43) | <0.001 |
|  | HR (95%CI) | *p* |  | HR (95%CI) | *p* |
| Low NfL |  |  |  |  |  |
| Without depressive symptoms | 1 (Ref) | - |  | 1 (Ref) | - |
| With depressive symptoms | 1.54 (0.63, 3.77) | 0.342 |  | 1.62 (0.61, 4.30) | 0.330 |
| High NfL |  |  |  |  |  |
| Without depressive symptoms | 1.71 (1.02, 2.85) | 0.041 |  | 1.59 (0.88, 2.86) | 0.127 |
| With depressive symptoms | 2.89 (1.48, 5.65) | 0.002 |  | 2.79 (1.27, 6.11) | 0.010 |

Note. IPCW-adjusted Cox regression model, adjusted for baseline age, years of education, APOE ε4, hypertension, hyperlipidemia, coronary heart disease, cigarette smoking, alcohol drinking, and BMI.

The low p-tau217, ≤ 0.42pg/mL; high p-tau217, > 0.42pg/mL. The low NfL, ≤ 15.95pg/mL; high p-tau217, > 15.95pg/mL. With Depressive symptoms, score of CES-D ≥ 16; Without Depressive symptoms, score of CES-D < 16.

Abbreviations: IPCW, inverse probability of censoring weighting; HR, hazard ratio; CI, confidence interval; NfL, neurofilament light chain; p-tau217, phosphorylated tau 217; AD, Alzheimer’s disease.

Sensitivity analysis: redefining depressive symptoms with CES-D ≥ 20

**Table S6.** Baseline and follow-up characteristics of participants by depressive symptoms (CES-D ≥ 20).

|  | Without depressive symptoms | With depressive symptoms | *p* |
| --- | --- | --- | --- |
|  | N=1519 | N=139 |  |
| **Baseline** |  |  |  |
| Age, years, Mean ± SD | 71.4 ± 7.4 | 72.3 ± 7.3 | 0.165 |
| Sex, male, n (%) | 708 (46.6) | 51 (36.7) | 0.031 |
| APOE ε4, positive, n (%) | 247 (16.3) | 26 (18.7) | 0.532 |
| Education, years, Mean ± SD | 11.9 ± 4.0 | 11.7 ± 4.1 | 0.467 |
| MMSE, Mean ± SD | 28.2 ± 2.0 | 27.8 ± 2.4 | 0.027 |
| Cigarette smoking, n (%) | 315 (20.7) | 19 (13.7) | 0.060 |
| Alcohol drinking, n (%) | 140 (9.2) | 9 (6.5) | 0.354 |
| BMI, Mean ± SD | 24.6 ± 3.5 | 24.4 ± 3.8 | 0.581 |
| Hypertension, n (%) | 812 (53.5) | 83 (59.7) | 0.184 |
| Hyperlipidemia, n (%) | 550 (36.2) | 59 (42.4) | 0.171 |
| Coronary heart disease, n (%) | 172 (11.3) | 22 (15.8) | 0.149 |
| NfL, pg/mL, Mean ± SD | 18.6 ± 12.0 | 21.8 ± 14.8 | 0.059 |
| p-tau217, pg/mL, Mean ± SD | 0.4 ± 0.3 | 0.5 ± 0.5 | 0.456 |
| **Follow-up** |  |  |  |
| Follow-up years, median (range) | 5.2 (0.9, 7.3) | 5.1 (1.1, 7.1) | 0.523 |
| MMSE, Mean ± SD | 26.7 ± 4.1 | 25.9 ± 4.3 | 0.017 |
| Incident Dementia, n | 143 (1.8/100) | 25 (3.5/100) | 0.001 |
| (rate/100 person-years) |  |  |  |
| Incident AD, n | 107 (1.4/100) | 17 (2.4/100) | 0.026 |
| (rate/100 person-years) |  |  |  |

Note. Continuous variables were compared with the Mann-Whitney U test, and categorical variables were compared with Pearson’s chi-squared test.

Abbreviations: SD, standard deviation; APOE, apolipoprotein E; MMSE, Mini-Mental State Examination; BMI, body mass index; NfL, neurofilament light chain; p-tau217, phosphorylated tau 217; AD, Alzheimer’s disease.

**Table S7.** Hazard ratios for incident dementia or AD with depressive symptoms (CES-D ≥ 20) and covariates.

|  | Dementia | |  | AD | |
| --- | --- | --- | --- | --- | --- |
|  | HR (95%CI) | *p* |  | HR (95%CI) | *p* |
| Depressive symptoms | 2.00 (1.29, 3.10) | 0.002 |  | 1.89 (1.12, 3.21) | 0.017 |
| Age | 1.16 (1.13, 1.19) | <0.001 |  | 1.16 (1.13, 1.19) | <0.001 |
| Female | 1.03 (0.69, 1.56) | 0.871 |  | 0.81 (0.50, 1.30) | 0.377 |
| Education years | 0.91 (0.88, 0.93) | <0.001 |  | 0.89 (0.85, 0.92) | <0.001 |
| Cigarette smoking | 1.15 (0.69, 1.90) | 0.592 |  | 0.99 (0.55, 1.77) | 0.970 |
| Alcohol drinking | 0.64 (0.31, 1.35) | 0.241 |  | 0.51 (0.20, 1.30) | 0.158 |
| BMI | 0.97 (0.92, 1.02) | 0.184 |  | 0.96 (0.91, 1.02) | 0.161 |
| Hypertension | 1.31 (0.93, 1.83) | 0.120 |  | 1.06 (0.72, 1.56) | 0.753 |
| Hyperlipidemia | 0.73 (0.52, 1.04) | 0.084 |  | 0.75 (0.50, 1.14) | 0.186 |
| Coronary heart disease | 1.20 (0.80, 1.80) | 0.382 |  | 1.01 (0.61, 1.67) | 0.962 |
| APOE ε4 | 1.36 (0.92, 2.00) | 0.120 |  | 1.52 (0.99, 2.33) | 0.055 |

Note. Multivariate Cox regression model, adjusted for age, sex, years of education, cigarette smoking, alcohol drinking, BMI, hypertension, hyperlipidemia, coronary heart disease and APOE ε4.

Abbreviations: HR, hazard ratio; CI, confidence interval; BMI, body mass index; APOE, apolipoprotein E.

**Table S8.** Hazard ratios for incident dementia and AD across subgroups in all participants.

|  | All (N=1658) | | | | |
| --- | --- | --- | --- | --- | --- |
|  | N | HR (95%CI) for Dementia | *p* | HR (95%CI) for AD | *p* |
| Low p-tau217 |  |  |  |  |  |
| Without depressive symptoms | 1076 | 1 (Ref) | - | 1 (Ref) | - |
| With depressive symptoms | 94 | 1.31 (0.63, 2.76) | 0.471 | 0.90 (0.32, 2.52) | 0.843 |
| High p-tau217 |  |  |  |  |  |
| Without depressive symptoms | 443 | 1.91 (1.33, 2.73) | <0.001 | 1.98 (1.31, 2.99) | 0.001 |
| With depressive symptoms | 45 | 5.10 (2.91, 8.94) | <0.001 | 5.57 (2.92, 10.62) | <0.001 |
|  | N | HR (95%CI) for Dementia | *p* | HR (95%CI) for AD | *p* |
| Low NfL |  |  |  |  |  |
| Without depressive symptoms | 763 | 1 (Ref) | - | 1 (Ref) | - |
| With depressive symptoms | 66 | 2.03 (0.79, 5.24) | 0.144 | 1.60 (0.48, 5.34) | 0.444 |
| High NfL |  |  |  |  |  |
| Without depressive symptoms | 756 | 1.65 (1.09, 2.50) | 0.018 | 1.57 (0.97, 2.52) | 0.064 |
| With depressive symptoms | 73 | 3.23 (1.80, 5.80) | <0.001 | 2.99 (1.50, 5.94) | 0.002 |

Multivariate Cox regression model, adjusted for baseline age, sex, years of education, APOE ε4, hypertension, hyperlipidemia, coronary heart disease, cigarette smoking, alcohol drinking, and BMI.

The low p-tau217, ≤ 0.42pg/mL; high p-tau217, > 0.42pg/mL. The low NfL, ≤ 15.95pg/mL; high p-tau217, > 15.95pg/mL. With Depressive symptoms, score of CES-D ≥ 20; Without Depressive symptoms, score of CES-D < 20.

Abbreviations: HR, hazard ratio; CI, confidence interval; NfL, neurofilament light chain; p-tau217, phosphorylated tau 217; AD, Alzheimer’s disease.

**Table S9.** Hazard ratios for incident dementia and AD across subgroups in men.

|  | Men (N=759) | | | | |
| --- | --- | --- | --- | --- | --- |
|  | N | HR (95%CI) for Dementia | *p* | HR (95%CI) for AD | *p* |
| Low p-tau217 | 505 | 1 (Ref) | - | 1 (Ref) | - |
| High p-tau217 |  |  |  |  |  |
| Without depressive symptoms | 233 | 1.15 (0.64, 2.08) | 0.637 | 0.86 (0.43, 1.72) | 0.673 |
| With depressive symptoms | 21 | 2.96 (1.06, 8.33) | 0.039 | 3.45 (1.17, 10.20) | 0.025 |
|  | N | HR (95%CI) for Dementia | *p* | HR (95%CI) for AD | *p* |
| Low NfL | 350 | 1 (Ref) | - | 1 (Ref) | - |
| High NfL |  |  |  |  |  |
| Without depressive symptoms | 377 | 1.19 (0.59, 2.40) | 0.618 | 1.27 (0.56, 2.90) | 0.575 |
| With depressive symptoms | 32 | 4.02 (1.47, 11.01) | 0.007 | 4.65 (1.48, 14.60) | 0.008 |

Multivariate Cox regression model, adjusted for baseline age, years of education, APOE ε4, hypertension, hyperlipidemia, coronary heart disease, cigarette smoking, alcohol drinking, and BMI.

The low p-tau217, ≤ 0.42pg/mL; high p-tau217, > 0.42pg/mL. The low NfL, ≤ 15.95pg/mL; high p-tau217, > 15.95pg/mL. With Depressive symptoms, score of CES-D ≥ 20; Without Depressive symptoms, score of CES-D < 20.

Abbreviations: HR, hazard ratio; CI, confidence interval; NfL, neurofilament light chain; p-tau217, phosphorylated tau 217; AD, Alzheimer’s disease.

**Table S10.** Hazard ratios for incident dementia and AD across subgroups in women.

|  | Women (N=899) | | | | |
| --- | --- | --- | --- | --- | --- |
|  | N | HR (95%CI) for Dementia | *p* | HR (95%CI) for AD | *p* |
| Low p-tau217 |  |  |  |  |  |
| Without depressive symptoms | 601 | 1 (Ref) | - | 1 (Ref) | - |
| With depressive symptoms | 64 | 1.44(0.60, 3.43) | 0.412 | 0.97 (0.29, 3.24) | 0.970 |
| High p-tau217 |  |  |  |  |  |
| Without depressive symptoms | 210 | 2.65 (1.68, 4.19) | <0.001 | 3.12 (1.82, 5.34) | <0.001 |
| With depressive symptoms | 24 | 7.00 (3.55, 13.79) | <0.001 | 6.89 (3.00, 15.80) | <0.001 |
|  | N | HR (95%CI) for Dementia | *p* | HR (95%CI) for AD | *p* |
| Low NfL |  |  |  |  |  |
| Without depressive symptoms | 432 | 1 (Ref) | - | 1 (Ref) | - |
| With depressive symptoms | 47 | 3.37 (1.24, 9.18) | 0.017 | 2.48 (0.71, 8.67) | 0.154 |
| High NfL |  |  |  |  |  |
| Without depressive symptoms | 379 | 2.32 (1.35, 3.98) | 0.002 | 1.88 (1.03, 3.43) | 0.040 |
| With depressive symptoms | 41 | 3.84 (1.83, 8.03) | <0.001 | 2.56 (1.05, 6.23) | 0.038 |

Multivariate Cox regression model, adjusted for baseline age, years of education, APOE ε4, hypertension, hyperlipidemia, coronary heart disease, cigarette smoking, alcohol drinking, and BMI.

The low p-tau217, ≤ 0.42pg/mL; high p-tau217, > 0.42pg/mL. The low NfL, ≤ 15.95pg/mL; high p-tau217, > 15.95pg/mL. With Depressive symptoms, score of CES-D ≥ 20; Without Depressive symptoms, score of CES-D < 20.

Abbreviations: HR, hazard ratio; CI, confidence interval; NfL, neurofilament light chain; p-tau217, phosphorylated tau 217; AD, Alzheimer’s disease.

Sensitivity analysis: using the median of p-tau217 as the cutoff

**Table S11.** Overall and sex-specific hazard ratios for incident dementia and AD across subgroups using the median of p-tau217 (0.32pg/mL) as the cutoff.

|  | All (N=1658) | | | | |
| --- | --- | --- | --- | --- | --- |
|  | N | HR (95%CI) for Dementia | *p* | HR (95%CI) for AD | *p* |
| Low p-tau217 |  |  |  |  |  |
| Without depressive symptoms | 713 | 1 (Ref) | - | 1 (Ref) | - |
| With depressive symptoms | 116 | 0.77 (0.30, 1.98) | 0.582 | 0.71 (0.21, 2.39) | 0.578 |
| High p-tau217 |  |  |  |  |  |
| Without depressive symptoms | 701 | 1.79 (1.18, 2.71) | 0.006 | 2.14 (1.29, 3.57) | 0.003 |
| With depressive symptoms | 128 | 3.52 (2.11, 5.88) | <0.001 | 4.43 (2.41, 8.12) | <0.001 |
|  | Men (N=759) | | | | |
|  | N | HR (95%CI) for Dementia | *p* | HR (95%CI) for AD | *p* |
| Low p-tau217 |  |  |  |  |  |
| Without depressive symptoms | 293 | 1 (Ref) | - | 1 (Ref) | - |
| With depressive symptoms | 37 | 0.72 (0.10, 5.68) | 0.759 | 1.01 (0.12, 8.29) | 0.991 |
| High p-tau217 |  |  |  |  |  |
| Without depressive symptoms | 368 | 1.12 (0.58, 2.19) | 0.739 | 1.11 (0.50, 2.47) | 0.793 |
| With depressive symptoms | 61 | 3.43 (1.47, 8.04) | 0.004 | 4.40 (1.68, 11.50) | 0.003 |
|  | Women (N=899) | | | | |
|  | N | HR (95%CI) for Dementia | *p* | HR (95%CI) for AD | *p* |
| Low p-tau217 |  |  |  |  |  |
| Without depressive symptoms | 420 | 1 (Ref) | - | 1 (Ref) | - |
| With depressive symptoms | 79 | 0.90 (0.31, 2.65) | 0.849 | 0.72 (0.16, 3.26) | 0.675 |
| High p-tau217 |  |  |  |  |  |
| Without depressive symptoms | 333 | 2.32 (1.36, 3.97) | 0.002 | 3.22 (1.64, 6.32) | <0.001 |
| With depressive symptoms | 67 | 3.43 (1.76, 6.70) | <0.001 | 4.24 (1.85, 9.73) | <0.001 |

Multivariate Cox regression model, adjusted for baseline age, years of education, APOE ε4, hypertension, hyperlipidemia, coronary heart disease, cigarette smoking, alcohol drinking, and BMI.

The low p-tau217, ≤ 0.32pg/mL; high p-tau217, > 0.32pg/mL. The low NfL, ≤ 15.95pg/mL; high p-tau217, > 15.95pg/mL. With Depressive symptoms, score of CES-D ≥ 16; Without Depressive symptoms, score of CES-D < 16.

Abbreviations: HR, hazard ratio; CI, confidence interval; p-tau217, phosphorylated tau 217; AD, Alzheimer’s disease.

Sensitivity analysis: incorporating the interaction terms sex*biomarker

**Table S12.** Hazard ratios of interaction terms for incident dementia and AD.

|  | Dementia | |  | AD | |
| --- | --- | --- | --- | --- | --- |
| Interaction term | HR (95%CI) | *p* |  | HR (95%CI) | *p* |
| p-tau217*sex | 0.62 (0.32, 1.18) | 0.143 |  | 0.43 (0.20, 0.90) | 0.026 |
| NfL*sex | 0.71 (0.31, 1.63) | 0.414 |  | 0.79 (0.30, 2.08) | 0.631 |

Multivariate Cox regression model, adjusted for baseline age, years of education, APOE ε4, hypertension, hyperlipidemia, coronary heart disease, cigarette smoking, alcohol drinking, BMI and sex*p-tau217/NfL.

Abbreviations: HR, hazard ratio; CI, confidence interval; NfL, neurofilament light chain; p-tau217, phosphorylated tau 217; AD, Alzheimer’s disease.


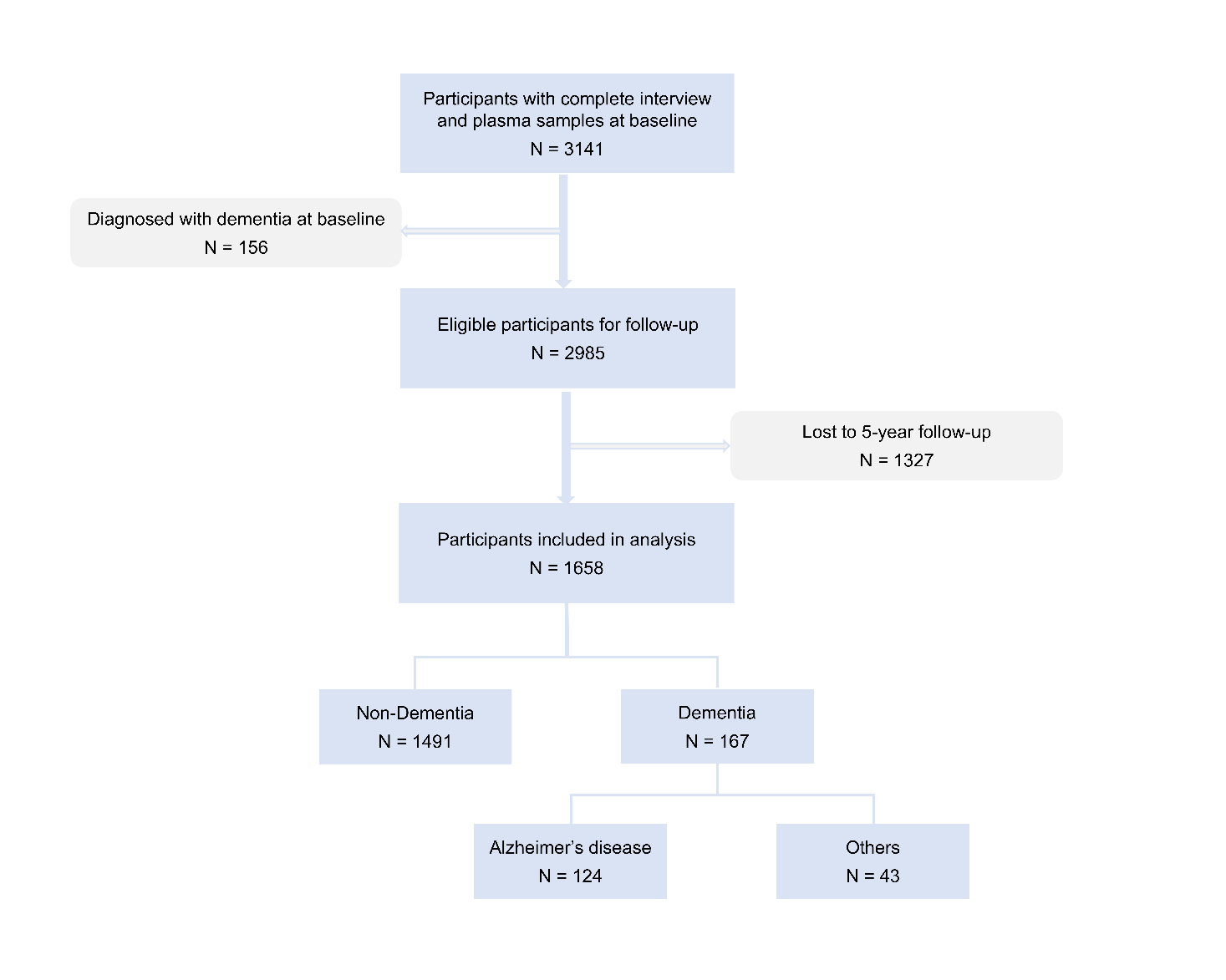


**Figure S1.** Flow chart of study participants.

Sensitivity analysis: using Cox-GAM models
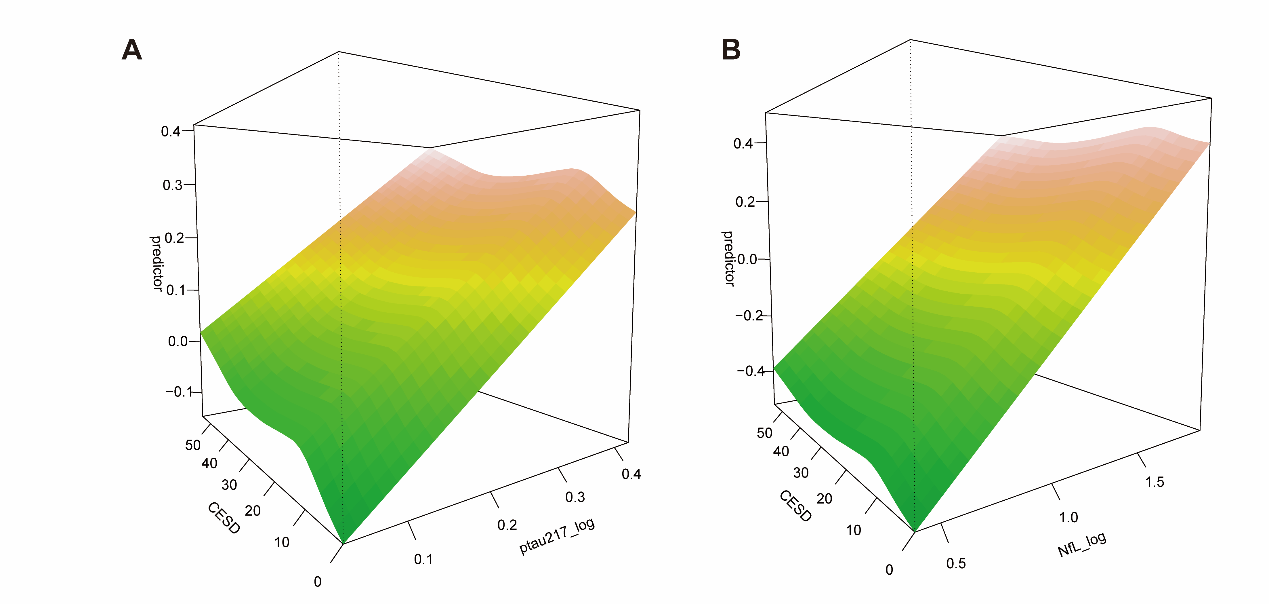


**Figure S2.** Predicted dose-response surface plots for the joint effects of baseline CES-D scores and p-tau217/NfL on dementia incidence.


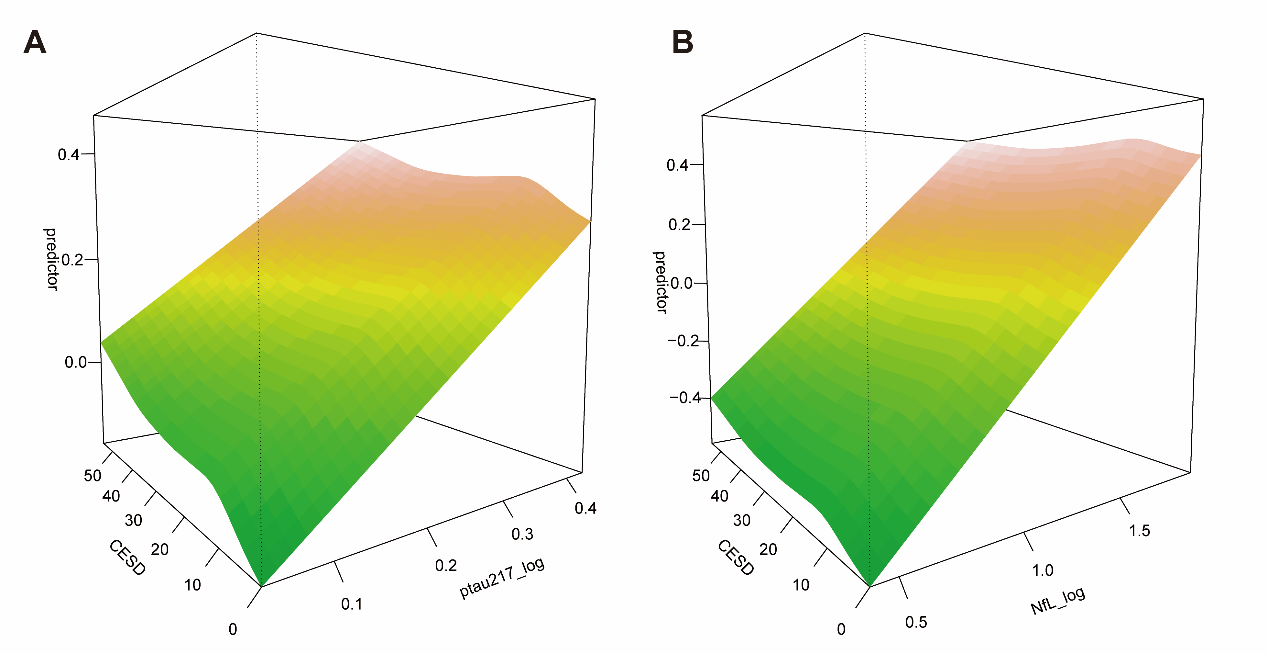


**Figure S3.** Predicted dose-response surface plots for the joint effects of baseline CES-D scores and p-tau217/NfL on AD incidence.
